# Higher number of steps and breaks during sedentary behaviour are associated with better lipid profiles

**DOI:** 10.1186/s12889-021-10656-5

**Published:** 2021-03-31

**Authors:** Sonja Aho, Meri-Sisko Vuoristo, Jani Raitanen, Kirsi Mansikkamäki, Johanna Alanko, Henri Vähä-Ypyä, Riitta Luoto, Pirkko-Liisa Kellokumpu-Lehtinen, Tommi Vasankari

**Affiliations:** 1 Faculty of Medicine and Health Technology, Tampere University and TAYS Cancer Center, Tampere University Hospital, Tampere, Finland; 2 Department of Oncology, Tampere University Hospital, P. O. Box 2000, 33521 Tampere, Finland; 3 The UKK Institute for Health Promotion Research, Tampere, Finland; 4 Tampere University, Faculty of Social Sciences (Health Sciences), Tampere, Finland; 5 Tampere University of Applied Sciences, Biomedical Laboratory Science, Tampere, Finland; 6 Tampere University, Faculty of Medicine and Health Technology, Tampere, Finland; 7 Tampere University Hospital, Research, Development and Innovation Center, Tampere, Finland

**Keywords:** Exercise, Breaks, Lipids, Physical Activity

## Abstract

**Background:**

Physical activity (PA) is known to be associated with lipid profiles and the risk of both cardiovascular diseases and cancer. The aim of this study was to evaluate the association of objectively measured PA, sedentary behaviour (SB), amount of breaks during SB and number of daily steps with serum lipids in a healthy, Finnish, middle-aged, female population.

**Methods:**

The participants (571) were recruited at mammography screening, target group was women aged 50–60 years. A measurement of PA was done with accelerometer, blood lipid profile was assessed, and questionnaires of participants characteristics were sent to participants.

**Results:**

The participants with the highest number of daily breaks during SB (≥ 41) had the highest mean concentration of HDL-cholesterol (high density lipoprotein cholesterol, HDL-c) (1.9 mmol/l, standard deviation (SD) 0.4) and the lowest mean concentration of triglycerides (1.0 mmol/l, SD 0.5). HDL-c level was 0.16 mmol/l higher (*p* < 0.001) in the group with 28–40.9 breaks/day and 0.25 mmol/l higher (*p* < 0.001) among participants with ≥41 breaks/day than in the group with the fewest breaks during SB (< 28).

Those with the most daily steps (≥ 9100) had the highest mean HDL-c level (1.9 mmol/l). HDL-c level was 0.16 mmol/l higher (*p* < 0.001) among the participants with 5600–9099 steps/day and 0.26 mmol/l higher (*p* < 0.001) among participants with ≥9100 steps/day than those with the fewest steps (< 5600). The number of daily steps was inversely associated with the triglyceride concentration.

From wake-time, participants spent 60% in SB, 18% standing, 14% in light PA, and 9% in moderate-to-vigorous PA (MVPA). PA was associated with serum total cholesterol (TC), HDL-c and triglyceride levels. The mean HDL-c level was the highest in the lowest quartile of SB and in the highest quartile of MVPA.

**Conclusions:**

To our knowledge, this is the first study showing a high number of objectively measured breaks during SB is associated with a favourable effect on the level of serum lipids, which may later translate into cardiovascular health among middle-aged women.

**Trial registration:**

This study was registered and approved by the Regional Ethics Committee of Tampere University Hospital in Finland (approval code R15137).

**Supplementary Information:**

The online version contains supplementary material available at 10.1186/s12889-021-10656-5.

## Background

Physical activity (PA) is defined as an energy expenditure of > 1.5 metabolic equivalents (METs) [1, 2]. Sedentary behaviour (SB) refers to lying down or sitting with an energy expenditure of ≤1.5 METs, and it has been recently identified as a risk factor for cardiovascular diseases (CVDs) independent from PA [3–6]. PA has also been associated with CVDs in several studies [1, 5]. Physical inactivity is an independent risk factor for CVDs [2]. A longer time spent in SB is associated with a larger waist circumference [7]. It has been suggested that a low number of breaks during SB is an independent risk factor for CVDs [8]. A recent publication suggests that the duration of SB and moderate to vigorous physical activity (MVPA) bouts should also be considered when determining one’s CVD risk [8]. Modern algorithms are capable of differentiating different types of sedentary behaviour and measuring the number and duration of breaks during sedentary time [9].

A recommendation recently published by the 2018 Physical Activity Guidelines Advisory Committee suggests that adults should perform moderate-intensity PA for at least 150 min to 300 min/week, vigorous-intensity aerobic PA for 75 min to 150 min/week, or an equivalent combination of moderate- and vigorous-intensity aerobic activity. The guidelines also suggest that adults should perform muscle-strengthening activities on > 2 days/week. Moving more and sitting less will benefit nearly all individuals [10].

CVDs are the leading cause of death globally [11]. CVDs are responsible for 32% of all deaths worldwide [11]. The main risk factors for CVDs are a genetic vulnerability, old age, male sex, obesity, smoking, a diet high in saturated fats, diabetes, hypertension, low physical activity and an atherogenic lipid profile [3, 4, 12]. Markers of an atherogenic lipid profile include low levels of high density lipoprotein cholesterol (HDL-c), high levels of low density lipoprotein cholesterol (LDL-c), total cholesterol (TC), triglycerides, and apolipoprotein (a), and high levels of oxidized LDL [13].

In addition, it has been shown that physical inactivity increases the risk of breast cancer, colorectal cancer (CRC) and possibly prostate cancer [14, 15]. There is evidence of an inverse relationship between physical activity, all-cause deaths, breast cancer-related deaths and breast cancer [16]. Obesity and physical inactivity are associated with poorer overall and disease-specific survival in patients with CRC [17]. Additionally, a high body mass index (BMI) in general is associated with a risk of developing cancer [18, 19].

Objective measurements of PA and SB can be performed by an accelerometer [1]. Objectively measured data are considered more reliable than self-reported data [20]. Accelerometers can determine the duration, intensity, number and frequency of accumulated PA and SB [1, 2].

This prospective study investigated the association of objectively measured PA, SB, number of daily steps and number of breaks during SB with serum TC, LDL-c, HDL-c and triglyceride levels among a healthy female middle-aged population participating in a breast cancer screening programme. To the best of our knowledge, an association between the device-measured number of breaks during SB and serum lipid profiles has not been previously reported.

## Materials and methods

### Study population

In this prospective cohort study, the participants were recruited from Tampere City Hospital Breast Clinic in Finland. Recruitment efforts were conducted one week per month for a total of 11 weeks. The target group for our study included women who were 50 to 60 years of age and participated in the breast cancer screening programme. Written information about this study was provided to all attendees. Those willing to participate returned the signed informed consent forms via mail.

**Fig. 1:**
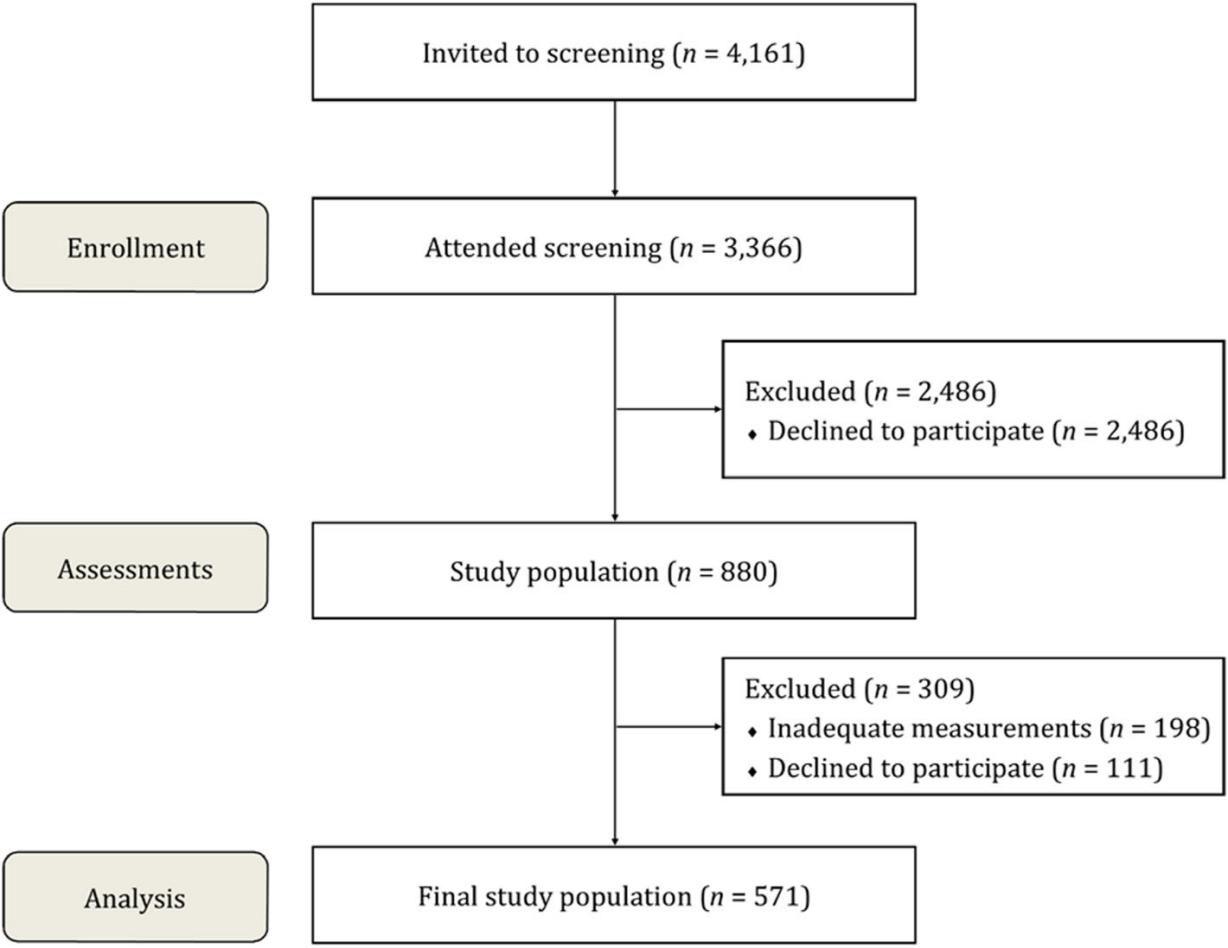
Flow chart

A total of 3366 women attended the screening programme during the 11 weeks. Of these women, 880 (26.1%) were willing to participate in the study (Fig. 1). The participants (*n* = 650,73.9%) wore an accelerometer on their waist for a 14-day (two weeks) data collection period. From the original group of women, 667 (75.6%) underwent the laboratory test. Finally, this study comprised of 571 (64.9%) women who met the criteria for sufficient accelerometer data (at least four days per week with 10 or more hours per day based on guidelines used in previous reports [1, 21]) and underwent the laboratory tests.

### Study design

Flow chart of the study population is presented in Fig. 1. An accelerometer, questionnaire (including items on comorbidities, medications, weight, height, smoking status and quality of life measured with the SF-36 and 15D, Appendix) and instructions for the laboratory tests were sent to the participants. Within two weeks the participants were instructed to visit laboratory for fasting venous blood samples. Plasma and serum was separated and stored at − 70° until analysed. The participants were randomized (1:1) to the intervention and control groups for long-term follow-ups. After the baseline measurements the participants received feedback of the analysis of the PA and SB collected by the accelerometer. In addition, the intervention group was given an exercise prescription. Further, the participants that were randomized at baseline, where invited to 1-year and 3-year follow-up measurements. Here, we report the baseline results before randomization.

### Measurement of physical activity (PA) and sedentary behaviour (SB)

PA and SB were measured at a 100 Hz sampling rate with tri-axial accelerometers (Hookie AM20 (Traxmeet Ltd., Espoo, Finland) and UKK AM30 (UKK Terveyspalvelut Oy, Tampere, Finland)) attached to a flexible belt that was worn on the right hip. The participants were given written instructions on how to use the accelerometer for 14 consecutive days during their waking hours. The accelerometer was not allowed to be worn during water activities, such as shower or swimming. The raw accelerometer data were stored on a mass storage device and sent back via mail to the research group for further analysis. All data were relocated to an analysing program written in Visual Basic for Applications to Microsoft Excel 2016 (Microsoft Corp. Redmond, WA, USA) and analysed according to the instructions described below.

We considered the mean amplitude deviation (MAD) values determined from the resultant acceleration of the three orthogonal acceleration components in six-second epochs as a valid indicator of incident oxygen consumption during movement [22, 23]. The MAD values were converted to the metabolic equivalent of task values (METs) (3.5 ml/kg/min of oxygen consumption) for each epoch. Intensity was calculated as the one-minute moving exponential average of the estimated MET values.

PA was classified into three intensity categories in terms of the METs: light PA (1.5–2.9 METs), moderate PA (3.0–5.9 METs and vigorous PA (more than 6.0 METs) [1]. Moderate and vigorous PA are combined with MVPA in many studies.

SB was defined as the time spent in a seated and/or resting position without movement (≤1.5 METs). Standing without movement was analysed separately. The classification of body posture was determined based on two facts: Earth’s gravity vector is constant, and the body’s posture while walking is upright. The accelerometer orientation with respect to the gravity vector was taken as the reference orientation, and the posture was determined from the incident accelerometer orientation with respect to the reference vector [9]. A transition from sitting to standing was considered a break in SB. The daily number of sit-to-stand transitions was based on the amount of lying/sitting periods during which the previous one minute estimated MET value indicated no movement and which ended up with a clear vertical acceleration followed by a standing position or movement [23]. The step detection algorithm split the measured acceleration data into vertical and horizontal components. The vertical component was bandpass filtered (1–4 Hz), and the positive values were integrated. When the integral value exceeded the specified limit, a step was detected [23].

### Blood samples

Peripheral blood were collected at baseline for analysis of the serum TC (mmol/l), HDL-c (mmol/l), LDL-c (mmol/l), and triglyceride levels (mmol/l). The study participants were instructed to fast for 10–12 h before the samples were collected. The samples were taken in the laboratory at the Pirkanmaa Cancer Society in Tampere, Finland [24] and transported to be analysed at the United Medix Laboratories Ltd. in Helsinki, Finland [25]. In this report, the analysis of the TC, HDL-c, LDL-c and triglyceride levels was performed by the enzymatic method. The normal ranges were as follows: < 5.0 mmol/l for TC, > 1.2 mmol/l for HDL-c, < 3.0 mmol/l for LDL-c and < 1.7 mmol/l for triglycerides.

### Statistical analysis

The study population was divided into quartiles based on the following:

1. the total number of daily breaks during SB,
2. the total number of daily steps,
3. the total time of SB, and
4. the total time of MVPA as a proportion of the total measurement time.

The two middle subgroups (Q2 and Q3) were combined for the statistical analysis so that it was possible to compare the highest quartile (Q4) and the middle group (Q2–3) to the lowest quartile (Q1). The cut-off points for the lowest and highest quartiles were 54 and 66%, respectively, which corresponded to 6.8 and 10.8% of the total time accumulated for SB and MVPA, respectively. The corresponding cut-off points were 28 and 41 for the daily breaks and 5600 and 9100 for the daily steps.

Descriptive statistics (means and standard deviations (SDs)) were reported to summarize the participants’ characteristics. Linear regression analysis was used to examine the relationship between SB, MVPA, number of daily breaks, and number of steps per day and the TC, HDL-c, LDL-c, and triglyceride levels, as well as the difference between the groups (Q1, Q2–3, and Q4) in the TC, HDL-c, LDL-c, and triglyceride levels. Log-transformation for triglycerides was used in the linear regression models to obtain normally distributed residuals. Both unadjusted models and models adjusted for the BMI, hypothyroidism medication use, and hormone replacement therapy were generated. For analysis of the serum TC and LDL-c, the participants using statin medication as a lipid lowering therapy (*n* = 29) and the participants who did not respond questions concerning lipid lowering medication (*n* = 33) were excluded (total *n* = 62).

Analyses were performed with SPSS Statistics 25 (IBM, Armonk, NY, USA), and a *p*-value < 0.05 was considered statistically significant.

## Results

### Baseline characteristics

The baseline characteristics of 571 participants are summarized in Table 1. Out of the 15 diabetic patients, 14 (2.5%) reported taking medications for diabetes. Only 86 (16%) of the participants reported using hormone replacement therapy. In addition, 66 (12%) used over-the-counter products for menopausal symptoms. Smoking was uncommon; 58 (11%) reported being occasional or daily smokers, and 477 (89%) were never smokers or had stopped smoking.

**Table 1:** Baseline characteristics, mean and (standard deviation)

Age (years)	53.1 (4.0)
Height (cm)	166.1 (6.6)
Weight (kg)	71.9 (13.2)
BMI (kg/m^2^)	26.0 (4.6)
Total cholesterol	5.30 (0.88)
HDL-c	1.78 (0.41)
LDL-c	3.00 (0.77)
Triglycerides	1.14 (0.57)
SB ^a^	59.7 (8.9)
Standing ^a^	17.6 (5.9)
Light PA ^a^	13.8 (3.9)
MVPA ^a^	8.9 (2.9)
Breaks (number/day)	34.8 (9.6)
Steps (number/day)	7483 (2682)
Current smoker	40 (7.5)
Hypertension	102 (19.3)
Antihypertensive medication	104 (19.3)
Statin treatment	29 (5.4)
Use of anticoagulants	12 (12.2)
Diabetes	15 (2.8)
Insulin treatment	4 (0.7)
Oral diabetes medications	10 (1.9)
Hypothyroidism with medication	67 (12.3)
Use of HRT	86 (16.0)

*n* = 533–571

*BMI* body mass index, *HDL-c* high-density lipoprotein cholesterol, *LDL-c* low-density lipoprotein cholesterol, *SB* sedentary behaviour, *PA* physical activity, *MVPA* mean to vigorous physical activity, *HRT* hormone replacement therapy

^a^ Proportion of the total measurement time

In the baseline physical activity measurements, the mean amount of SB was 60% (SD 8.9%) of the total measurement time. From the total measured time, the participants spent 18% (SD 5.9%) standing, 14% (SD 3.9%) performing light PA, and 8.9% (SD 2.9%) performing MVPA in addition to the 60% of SB. On average, the participants took 7483 steps daily (SD 2682), and they had 35 (SD 9.6) breaks during sedentary behaviour (Table 1).

### Linear regression analysis of physical activity types and lipids

According to the linear regression analysis, light physical activity was associated with the concentration of HDL-c (coefficient 0.019, *p* < 0.001) (Table 2). After the models were adjusted for BMI, hypothyroidism medication use and hormone replacement therapy (HRT), this association remained still significant (coefficient 0.011, *p* = 0.005). The light PA was inversely related to the concentration of triglycerides in the unadjusted model (coefficient − 0.011, *p* = 0.012), but this association disappeared after the adjustments were made (coefficient − 0.004, *p* = 0.40) (Table 2). MVPA was associated with HDL-c both in the unadjusted model (coefficient 0.033, *p* < 0.001) and adjusted models (coefficient 0.018, *p* = 0.001) (Table 2). In addition, MVPA was inversely associated with the triglyceride concentration (coefficient − 0.017, *p* = 0.004), but this association disappeared after the model was adjusted for BMI, hypothyroidism medication use and HRT (coefficient − 0.004, *p* = 0.49) (Table 2). The number of breaks in SB was also associated with the concentration of HDL-c both in the unadjusted (coefficient 0.010, *p* < 0.001) and adjusted models (coefficient 0.005, *p* = 0.007). Breaks were associated with the triglyceride concentration in the unadjusted model (coefficient − 0.009, *p* < 0.001) and in the adjusted model (coefficient − 0.004, *p* = 0.028) (Table 2). The number of daily steps was associated with the concentration of HDL-c both in the unadjusted (coefficient 0.037, *p* < 0.001) and in the adjusted models (coefficient 0.019, *p* = 0.002). Additionally, the number of daily steps was inversely associated with the triglyceride concentration in the unadjusted model (coefficient − 0.024, *p* < 0.001), but this association disappeared after the model was adjusted for the BMI, hypothyroidism medication use and HRT (coefficient − 0.008, *p* = 0.23) (Table 2).

**Table 2:** Regression coefficients with their 95% confidence intervals (CIs) from the linear regression models predicting lipids

	Unadjusted			Adjusted^a^			
	*n*	Coeff. (95% CI)	*p*	*n*	Coeff. (95% CI)	*p*	
Laying down and sitting							
s-cholesterol	506	−0.004 (− 0.012 to 0.004)	0.34	501	− 0.004 (− 0.013 to 0.005)	0.34	
HDL-c	568	−0.013 (− 0.017 to − 0.010)	< 0.001	530	−0.006 (− 0.010 to − 0.003)	0.001	
LDL-c	509	0.003 (− 0.004 to 0.010)	0.43	504	− 0.000 (− 0.008 to 0.008)	0.99	
Triglycerides	568	0.010 (0.006 to 0.013)	< 0.001	530	0.003 (−0.000 to 0.007)	0.087	
Standing upright							
s-cholesterol	506	−0.007 (− 0.020 to 0.005)	0.25	501	−0.009 (− 0.022 to 0.005)	0.19	
HDL-c	568	0.014 (0.009 to 0.020)	< 0.001	530	0.004 (− 0.002 to 0.009)	0.20	
LDL-c	509	−0.014 (− 0.025 to − 0.003)	0.016	504	−0.011 (− 0.023 to 0.001)	0.072	
Triglycerides	568	−0.013 (− 0.019 to − 0.008)	< 0.001	530	− 0.005 (− 0.011 to 0.001)	0.13	
Light physical activity							
s-cholesterol	506	0.019 (0.000 to 0.038)	0.045	501	0.019 (0.000 to 0.038)	0.050	
HDL-c	568	0.019 (0.011 to 0.027)	< 0.001	530	0.011 (0.003 to 0.019)	0.005	
LDL-c	509	0.010 (−0.007 to 0.027)	0.24	504	0.013 (−0.004 to 0.030)	0.14	
Triglycerides	568	−0.011 (− 0.020 to − 0.002)	0.012	530	−0.004 (− 0.012 to 0.005)	0.40	
MVPA							
s-cholesterol	506	0.033 (0.008 to 0.058)	0.009	509	0.035 (0.009 to 0.061)	0.008	
HDL-c	568	0.033 (0.022 to 0.044)	< 0.001	530	0.018 (0.007 to 0.029)	0.001	
LDL-c	509	0.011 (−0.012 to 0.034)	0.35	504	0.018 (−0.005 to 0.042)	0.12	
Triglycerides	568	−0.017 (− 0.029 to − 0.005)	0.004	530	−0.004 (− 0.016 to 0.007)	0.49	
Number of breaks per day							
s-cholesterol	506	0.007 (−0.000 to 0.015)	0.064	501	0.008 (−0.001 to 0.016)	0.055	
HDL-c	568	0.010 (0.007 to 0.013)	< 0.001	530	0.005 (0.001 to 0.008)	0.007	
LDL-c	509	0.003 (− 0.004 to 0.010)	0.44	504	0.006 (−0.002 to 0.013)	0.12	
Triglycerides	568	−0.009 (− 0.012 to − 0.005)	< 0.001	530	− 0.004 (− 0.008 to − 0.000)	0.028	
Number of steps (thousands) per day							
s-cholesterol	506	0.018 (− 0.009 to 0.046)	0.19	501	0.021 (− 0.008 to 0.049)	0.16	
HDL-c	568	0.037 (0.025 to 0.049)	< 0.001	530	0.019 (0.007 to 0.031)	0.002	
LDL-c	509	−0.003 (− 0.027 to 0.022)	0.84	504	0.007 (−0.019 to 0.033)	0.58	
Triglycerides	568	−0.024 (− 0.037 to − 0.012)	< 0.001	530	−0.008 (− 0.020 to 0.005)	0.23	

*HDL-c* high density lipoprotein cholesterol, *LDL-c* low density lipoprotein cholesterol, *Triglycerides* log-transformed triglyceride concentration, *MVPA* moderate-to-vigorous physical activity

^a^ Adjusted for BMI, hypothyroidism medication use, and hormone replacement therapy. In all analyses concerning s-cholesterol and LDL-c the participants using statin therapy were excluded

The relationship between standing and LDL-c was significant in the unadjusted model (coefficient − 0.014, *p* = 0.016) but not in the adjusted model (− 0.011, *p* = 0.072) (Table 2). MVPA and light PA were associated with TC both in the unadjusted model (coefficient 0.033, *p* = 0.009 and coefficient 0.019, *p* = 0.045, respectively) and adjusted models (coefficient 0.035, *p* = 0.008 and coefficient 0.019, *p* = 0.050, respectively).

### Association of lipids and sedentary behaviour

The mean HDL-c level was the highest (1.92 (SD 0.43) mmol/l) in the participants who had the least amount of SB (SB less than 54% of waking hours) (Table 3).

**Table 3:** Serum lipid mean and standard deviations for the subgroups of participants grouped by the amounts of sedentary behaviour and moderate-to-vigorous physical activity performed

	SB			MVPA		
	< 54%	54–65.9%	≥ 66%	< 6.8%	6.8–10.79%	≥ 10.8%
s-cholesterol	5.40 (0.93)	5.30 (0.88)	5.19 (0.80)	5.17 (0.88)	5.28 (0.83)	5.46 (0.95)
HDL-c	1.92 (0.43)	1.80 (0.39)	1.60 (0.36)	1.64 (0.39)	1.79 (0.40)	1.90 (0.39)
LDL-c	3.00 (0.78)	3.00 (0.80)	3.00 (0.68)	2.97 (0.78)	2.99 (0.72)	3.06 (0.84)
Triglycerides	1.02 (0.51)	1.11 (0.58)	1.32 (0.59)	1.26 (0.59)	1.12 (0.57)	1.07 (0.55)

*SB* sedentary behaviour, *MVPA* moderate-to-vigorous physical activity, *HDL-c* high-density lipoprotein cholesterol, *LDL-c* low-density lipoprotein cholesterol

**Fig. 2:**
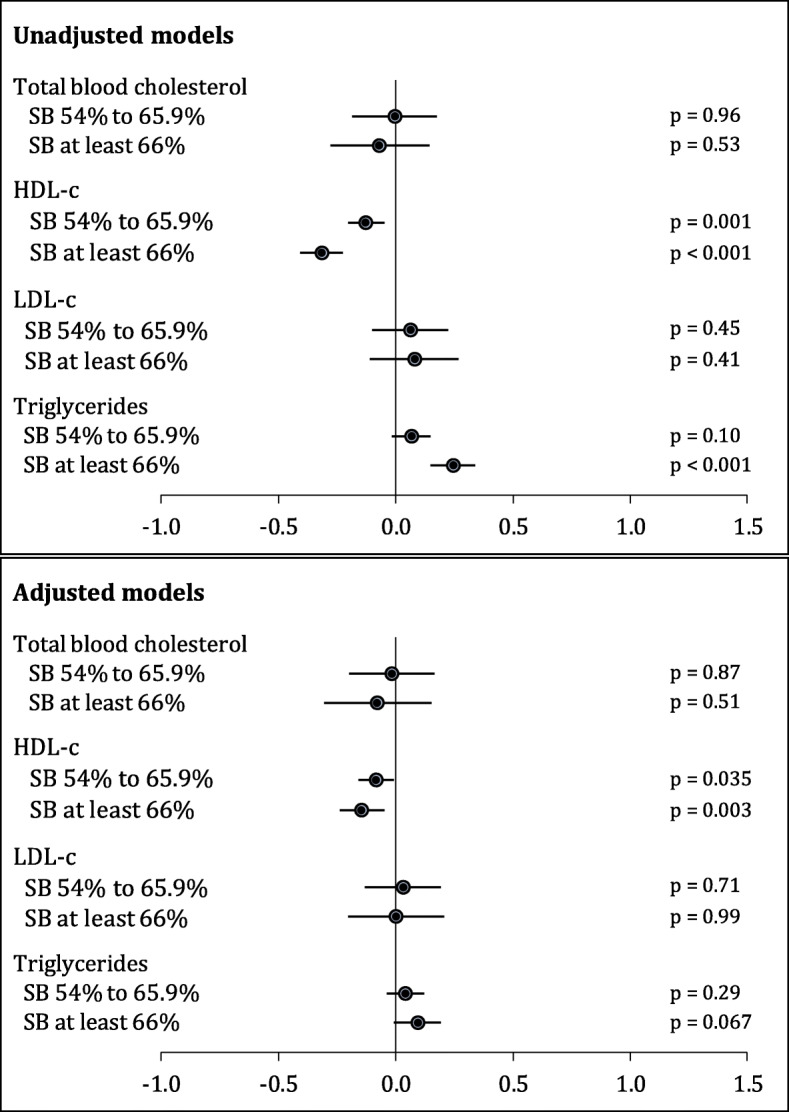
Regression coefficients with their 95% confidence intervals (CIs) from the linear regression models predicting lipids by the amount of sedentary behaviour (SB; &lt; 54% used as the reference group). HDL-c: high-density lipoprotein; LDL-c: low-density lipoprotein. Adjusted for BMI, hypothyroidism medication use, and hormone replacement therapy. In all analyses concerning s-cholesterol and LDL-c the participants using statin therapy were excluded

The triglyceride concentration was on average 0.25 mmol/l (*p* < 0.001) lower in the group with SB ≥ 66% than in the group of women with SB less than 54% (Fig. 2). The differences between the groups disappeared after the adjustments were made to the model (Fig. 2). Compared to the SB < 54% group, the SB 54–65.9% group and SB ≥ 66% group had lower HDL-c concentrations by 0.12 mmol/l (*p* = 0.001) and 0.32 mmol/l (*p* < 0.001), respectively (Fig. 2). The differences between the groups remained significant after the model was adjusted: the concentration of HDL-c was 0.08 mmol/l lower in the SB 54–65.9% group (*p* = 0.035) and 0.14 mmol/l lower in the SB ≥ 66% group (*p* = 0.003) than in the SB <  54% group (Fig. 2).

### Association of lipids and breaks during sedentary behaviour

When the number of daily breaks during SB were distributed to quartiles the mean concentration of HDL-c was the highest (1.89 mmol/l, SD 0.38), and the mean concentration of triglycerides was lowest (1.04 mmol/, SD 0.48) in the quartile with the most daily breaks (≥ 41) compared to the quartile with the lowest number of breaks (< 28).

In the unadjusted models, the HDL-c level was 0.16 mmol/l higher (*p* < 0.001) in the intermediate group (28–40.9 breaks) and 0.25 mmol/l higher (*p* < 0.001) in the group with the most breaks (≥ 41) than in the group with the fewest breaks during SB (< 28). After the model was adjusted for BMI, hypothyroidism medication use and HRT, the HDL-c level was 0.11 mmol/l higher (*p* = 0.017) in the group with ≥41 daily breaks during SB than in the group with < 28 breaks (Table 4).

**Table 4:** Regression coefficients with their 95% confidence intervals (CIs) from the linear regression models predicting lipids by the number of breaks in a day (fewer than 28 breaks in a day used as the reference group)

	Unadjusted				Adjusted^a^			
	Breaks 28–40.9 / day		Breaks 41+ / day		Breaks 28–40.9 / day		Breaks 41+ / day	
	Coeff. (95% CI)	*p*	Coeff. (95% CI)	*p*	Coeff. (95% CI)	*p*	Coeff. (95% CI)	*p*
s-cholesterol	0.10 (−0.08 to 0.28)	0.27	0.19 (−0.02 to 0.39)	0.070	0.10 (− 0.08 to 0.29)	0.28	0.21 (− 0.10 to 0.42)	0.062
HDL-c	0.16 (0.08 to 0.24)	< 0.001	0.25 (0.16 to 0.34)	< 0.001	0.06 (−0.02 to 0.14)	0.13	0.11 (0.02 to 0.20)	0.017
LDL-c	0.06 (−0.10 to 0.23)	0.44	0.09 (−0.10 to 0.27)	0.35	0.10 (−0.06 to 0.27)	0.23	0.16 (−0.03 to 0.36)	0.093
Triglycerides	−0.15 (− 0.24 to − 0.07)	0.001	−0.22 (− 0.31 to − 0.13)	< 0.001	−0.09 (− 0.17 to − 0.01)	0.031	−0.10 (− 0.20 to − 0.01)	0.032

*HDL-c* high-density lipoprotein cholesterol, *LDL-c* low-density lipoprotein cholesterol, *Triglycerides* log-transformed triglyceride concentration

^a^ Adjusted for BMI, hypothyroidism medication use, and hormone replacement therapy

In analyses concerning s-cholesterol and LDL-c the participants using statin therapy were excluded

The concentration of triglycerides was significantly lower both in the group with 28–40.9 breaks (− 0.15 mmol/l, *p* = 0.001) and in the group with ≥41 breaks (− 0.22 mmol/l, *p* < 0.001) than in the group with the fewest breaks (< 28). The differences remained significant even after the adjustments were made; the concentration of triglycerides was − 0.09 mmol/l lower (*p* = 0.031) in the group with 28–40.9 breaks and − 0.10 mmol/l lower (*p* = 0.032) in the group with ≥41 breaks when compared to the group with the fewest breaks (< 28) (Table 4).

### Association of lipids and daily steps

The mean number of steps per day for the whole study population was 7483 (SD 2682). In the group with the fewest steps (< 5600 steps/day), the mean concentration of HDL-c was 1.63 (SD 0.36) mmol/l. In the intermediate group (5600–9099 steps/day), the mean HDL-c was 1.80 (SD 0.41) mmol/l. The group with the most daily steps (≥ 9100) had the highest mean HDL-c level, which was 1.90 (SD 0.41) mmol/l.

In the unadjusted models, the HDL-c level was 0.16 mmol/l higher (*p* < 0.001) in the 5600–9099 steps/day group and 0.26 mmol/l higher (*p* < 0.001) in the ≥9100 steps/day group than in the group with the fewest steps (< 5600). After the model was adjusted for BMI, hypothyroidism medication use, and HRT, the HDL-c level was 0.13 mmol/l higher (*p* = 0.006) in the ≥9100 steps/day group than in the < 5600 steps/day group (Table 5).

**Table 5:** Regression coefficients with their 95% confidence intervals (CIs) from the linear regression models predicting lipids by the number of steps in day (fewer than 5600 steps in a day used as the reference group)

	Unadjusted				Adjusted^a^			
	5600–9099 steps / day		≥ 9100 steps / day		5600–9099 steps / day		≥ 9100 steps / day	
	Coeff. (95% CI)	*p*	Coeff. (95% CI)	*p*	Coeff. (95% CI)	*p*	Coeff. (95% CI)	*p*
s-cholesterol	0.06 (−0.12 to 0.25)	0.49	0.16 (−0.05 to 0.37)	0.13	0.09 (−0.10 to 0.28)	0.37	0.18 (−0.04 to 0.40)	0.10
HDL-c	0.16 (0.08 to 0.24)	< 0.001	0.26 (0.17 to 0.35)	< 0.001	0.04 (−0.03 to 0.12)	0.26	0.13 (0.04 to 0.22)	0.006
LDL-c	0.01 (−0.15 to 0.18)	0.87	0.02 (−0.17 to 0.20)	0.85	0.08 (−0.08 to 0.25)	0.33	0.10 (−0.10 to 0.29)	0.32
Triglycerides	−0.16 (− 0.24 to − 0.07)	< 0.001	−0.19 (− 0.29 to − 0.10)	< 0.001	−0.07 (− 0.15 to 0.10)	0.085	−0.08 (− 0.17 to 0.02)	0.10

*HDL-c* high-density lipoprotein cholesterol, *LDL-c* low-density lipoprotein cholesterol, *Triglycerides* log-transformed triglyceride concentration

^a^ Adjusted for BMI, hypothyroidism medication use, and hormone replacement therapy

In analyses concerning s-cholesterol and LDL-c the participants using statin therapy were excluded

The concentration of triglycerides was significantly lower in the groups with 5600–9099 (− 0.16 mmol/l, *p* < 0.001) and at least 9100 (− 0.19 mmol/l, *p* < 0.001) daily steps than in the group with fewer than 5600 daily steps. These significant findings disappeared after the adjustments were made (Table 5).

### Association of lipids and MVPA

As expected, the mean HDL-c level was the highest in the quartile with the highest amount of MVPA (at least 10.8% of waking hours). In this group, the mean HDL-c level was 1.90 (SD 0.39) mmol/l (Table 3). In the unadjusted linear regression models, the HDL-c level was 0.14 mmol/l (*p* = 0.001) higher in the MVPA 6.8–10.79% group and 0.25 mmol/l (*p* < 0.001) higher in the MVPA ≥10.8% group than in the MVPA less than 6.8% group. After the adjustments were made, the concentration of HDL-c was 0.14 mmol/l (*p* = 0.002) higher in the MVPA ≥10.8% group than in the MVPA < 6.8% group. In the unadjusted models, the concentration of triglycerides was 0.12 mmol/l (*p* = 0.005) lower in the MVPA 6.8–10.79% group and 0.15 mmol/l (*p* = 0.002) lower in the MVPA ≥10.8% group than in the MVPA < 6.8% group (Table 6). All these significant differences disappeared after the adjustments were made (Table 6).

**Table 6:** Regression coefficients with their 95% confidence intervals (CIs) from the linear regression models predicting lipids by the amount of moderate-to-vigorous physical activity (MVPA; < 6.8% used as the reference group)

	Unadjusted				Adjusted^a^			
	MVPA 6.8–10.79%		MVPA ≥10.8%		MVPA 6.8–10.79%		MVPA ≥10.8%	
	Coeff. (95% CI)	*p*	Coeff. (95% CI)	*p*	Coeff. (95% CI)	*p*	Coeff. (95% CI)	*p*
s-cholesterol	0.07 (−0.11 to 0.25)	0.42	0.25 (0.04 to 0.45)	0.019	0.10 (−0.09 to 0.28)	0.31	0.27 (0.05 to 0.48)	0.014
HDL-c	0.14 (0.06 to 0.22)	0.001	0.25 (0.16 to 0.34)	< 0.001	0.05 (−0.03 to 0.12)	0.24	0.14 (0.05 to 0.23)	0.002
LDL-c	0.03 (−0.14 to 0.19)	0.74	0.06 (−0.12 to 0.25)	0.50	0.09 (−0.08 to 0.26)	0.30	0.13 (−0.06 to 0.32)	0.17
Triglycerides	−0.12 (− 0.20 to − 0.04)	0.005	−0.15 (− 0.24 to − 0.05)	0.002	−0.04 (− 0.12 to 0.04)	0.35	−0.04 (− 0.13 to 0.06)	0.43

*HDL-c* high-density lipoprotein cholesterol, *LDL-c* low-density lipoprotein cholesterol, *Triglycerides* log-transformed triglyceride concentration

^a^ Adjusted for BMI, hypothyroidism medication use, and hormone replacement therapy

In analyses concerning s-cholesterol and LDL-c the participants using statin therapy were excluded

## Discussion

To the best of our knowledge, this study is the first to investigate the association of the number of breaks taken during SB, measured objectively by a device, and serum lipid profiles in a healthy middle-aged female population. Previous studies have focused on PA and SB in child [26, 27], youth [28] or adult populations with certain physiological states or illnesses [29–31] or adults with different types of professions [32, 33].

The quartile that had highest number of breaks per day had 0.11 mmol/l higher concentration of HDL-c and 0.10 mmol/l lower concentration of triglycerides compared to the quartile with lowest number of breaks in the adjusted model. Thus, participants in the quartile with the most breaks during SB had healthier lipid profiles than the quartile with the fewest breaks. This result might suggest better cardiovascular health in the future for the individuals with more breaks during SB. Therefore, it is hypothesized that in addition to the known positive effect of physical activity, our novel finding of the high amount of breaks during SB will have an impact on an individual’s CVD risk.

The mean amount of SB was higher in middle-aged women (60% of waking hours) than in the youth (53% of waking hours). However, these middle-aged women had more MVPA (8.9%) than the youth (5.3%) [28]. The association of the amount of MVPA and the concentration of HDL-c was similar in our middle-aged women as was shown in pregnant women [29].

The present study population represents the Finnish middle-aged female population well since the relevant baseline characteristics (BMI, age, TC, use of medications) are very similar to the Health 2011 study’s female population, which includes thousands of Finnish women [8, 34]. Thus, our results can be applied to the general population. Intervention studies on lifestyle changes in women invited to breast cancer screenings are ongoing, but they lack objectively measured PA data [35]. In addition, interventional studies on breast cancer patients showing a better quality of life among patients with the most physical activity have been published [36].

Participants in the present study took an average of 7483 steps per day. This result is in line with those reported in other accelerometer studies [8]. It seems that important cardiometabolic improvements emerge when people take > 7500 daily steps, and it is suggested that one should aim for at least 7500 daily steps and 150 min of MVPA per week [37]. In this study the quartile with the most steps had a significantly higher concentration of HDL-c than the quartile with the fewest daily steps.

According to the current 2020 ESC Guidelines on sports cardiology and exercise in patients with cardiovascular disease, physical activity has favourable effects on lipid metabolism by reducing serum triglycerides by up to 50% and increasing HDL-cholesterol by 5–10% [38]. Our study report that the quartiles with the highest number of breaks, the highest amount of MVPA, the most daily steps, and the least amount of SB had on average 0.1–0.3 mmol/l higher concentration of HDL-cholesterol and lower concentration of serum triglycerides than the corresponding least active quartiles. The current results support ESC Guidelines underlining the clinical message that high amount of PA and low amount of SB could be highly beneficial therapy for patients with unhealthy lipid profile.

In Finland, all women aged 50–69 years are invited by personal letters to participate in breast cancer screenings with mammography tests biennially, free of charge. Participation is voluntary. However, the participation rate has been high since the implementation of nationwide screening in 1987. In 2017, the nationwide participation rate for the breast cancer screening programme was 82%. In addition, 3% of participants were invited for further testing, i.e., ultrasound examinations and biopsies, and 0.6% were diagnosed with breast cancer [39]. This type of screening would offer a unique platform for lifestyle counselling for middle-aged women.

CVDs are very common in Finland. They account for 34% of deaths annually in Finland. In 2012 nearly 22,000 cases of myocardial infarction and coronary artery disease episodes were reported. Almost half of them in females [40]. It is therefore important to investigate ways to influence cardiovascular health in our population.

### Study limitations

The limitation of this type of study is that the participants are likely to comprise healthy volunteers who are already interested in their health. The volunteers are also likely to be more physically active. Altogether, 4161 women were invited for screenings with mammograms during the weeks we recruited study participants. Of these women, 3366 (81%) attended a screening, and 880 (26%) were willing to participate in this study. The mean BMI of the study population was slightly lower (26 kg/m^2^ (SD 4.6)) than in the FinHealth2017 Survey population study with the mean BMI of 28 kg/m^2^ among Finnish women in 2017 [41].

The recruitment method for the study was not very intensive. There were posters and flyers about the study in the screening mammography waiting room. Nurses at the screening facility mentioned about the recruiting process if they had the time to do so. This explains the loss of many participants and why the ones who wanted to participate are already quite physically active. However, there were 880 participants in this study, which is a reasonable amount.

The accelerometers were instructed to be used during waking hours and not during water-based activities. Therefore, some of the PA data were lost from the measurement period. However, the data are more objective and reliable than data in previous studies because these data are based on objective measurements and not on questionnaires only [42].

We lost a few participants and their data because a mail workers’ strike occurred during our study period. Because of the postal delay in the delivery, the battery for the accelerometers did not last for the whole measurement period.

Dietary behaviour could be a confounding factor in studies measuring serum lipids. In the present study we tried to control this important issue by collecting the participants from the breast cancer screening, which have a high participation rate in order to get normal population using different type of diet involved. Acute dietary effects were controlled by using overnight fasting before blood samples were taken.

The study data from the questionnaires are not objective. It might be tempting for the participants to embellish their responses, e.g., their weight, height or smoking status. Menopausal status was not addressed in our questionnaire, but due to the study’s target age group (50–60 years), it is likely that most of the participants were postmenopausal.

## Conclusions

Our prospective study with objective measurements of PA, SB, breaks during SB and the number of daily steps showed that a low volume of SB and a high number of breaks during SB and a greater amount of daily steps are associated with lower concentrations of triglycerides and higher concentrations of HDL-c in a healthy middle-aged female population. Our novel finding is that a greater number of breaks during SB and a higher number of daily steps are associated with favourable lipid profile.

### Availability of data and material

The data sets used and/or analysed during the current study are available from the corresponding author upon reasonable request.

## Supplementary Information

**Additional file 1.** Questionnaire. Questionnaire form. The parts of the questionnaire concerning background and lifestyle factors were developed for this study. Our questionnaire included generally used Health Related Quality of Life Questionnaires (SF-36 and 15-D), and the depression screening tool (DEPS) which are validated questionnaires and previously published in English in many studies of lifestyle factors and different type of diseases.

## Abbreviations

BMI: Body mass index
CVD: Cardiovascular disease
HDL-c: High density lipoprotein cholesterol
HRT: Hormone replacement therapy
LDL-c: Low density lipoprotein cholesterol
MAD: Mean amplitude deviation
MET: Metabolic equivalent of task
MVPA: Moderate to vigorous physical activity
PA: Physical activity
SB: Sedentary behaviour
SD: Standard deviation
TC: Total cholesterol
