# Supplementary material for: Higher number of steps and breaks during sedentary behaviour are associated with better lipid profiles
Source: BMC Public Health. 2021 Mar 31;21:629. doi: 10.1186/s12889-021-10656-5 (PMC8010961; doi:10.1186/s12889-021-10656-5)
Supplement: Supplementary file 1 — Additional file 1. Questionnaire. Questionnaire form. The parts of the questionnaire concerning background and lifestyle factors were developed for this study. Our questionnaire included generally used Health Related Quality of Life Questionnaires (SF-36 and 15-D), and the depression screening tool (DEPS) which are validated questionnaires and previously published in English in many studies of lifestyle factors and different type of diseases. [file 12889_2021_10656_MOESM1_ESM.docx]

***Date*** ­­­­­­­­­ _______________

***Identifier code***  __________________

**Women's Health Research – Hatanpää Breast Cancer Screening**

The aim of this questionnaire is to cover/evaluate lifestyle factors, illnesses, medical history, symptoms, use of health care services, ability to work, pregnancies and childbirths, mood and quality of life.

**Background factors**

1. ***Marital status***

1.1 Married or cohabitation /domestic partneship

1.2 Unmarried

1.3 Separated or divorced

1. 4 Widow

1. ***Education***
   1. Primary, secondary school or elementary school
   2. Matriculation examination/Baccalaureate
   3. Vocational school or initial vocational qualification
   4. Postsecondary vocational qualification
   5. Polytechnic or bachelor's degree
   6. Higher academic degree of the university
2. ***Your current profession***

–––––––––––––––––––––––––––––––––––––––––––––––––––––––––––––––––––––

1. **Do you have a disease or disability that hinders your ability to work and function?**

1 Yes

2 No

1. **If yes, What or what kind of disease or injury is it?**

––––––––––––––––––––––––––––––––––––––––––––––––––––––––––––––––––––––––––––––––––––––––––––––––––––––––––––––––––––––––––––––––––––––––––

**Cardiac diseases**

**Have you had**?

**6. Heart blood clot, or myocardial infarction?** 1 Yes 2 No

**7. Heart failure/insuffience?** 1 yes, 2 no

**8. Cardiac arrhythmia?** 1 yes, 2 no

**9. Any other heart condition or disease?** 1 yes, 2 no

a) 1 congenital heart defect

b) 2 heart valve failure

c) 3 heart nerve defects

d) 4 any other heart condition or disease?

**10. Hypertension?** 1 yes, 2 no

**11.Blood clot in the vein of the lower or upper limb (vein thrombous)?** 1 yes, 2 no

**12. Stroke (cerebral haemorrhage, cerebral blood clot)?** 1 yes, 2 no

**13. Arterial thrombosis of the lower limb or arterial stenosis?** 1 yes, 2 no

**Defects in joints, limbs and back, previous fractures and traumas**

**14. Rheumatoid arthritis?** 1 yes, 2 no

**15. Osteoarthrosis?** 1 yes, 2 no

**16. Other joint disease?** 1 yes, 2 no

**17. Disease of the back or other back defect?** 1 yes, 2 no

**18. Bone fracture or fractures?**  1 yes, 2 no where_______________________________________________________________

**19. Osteoporosis (loss of bone density)?** 1 yes, 2 no

Was it detected:

1 in X-ray

2 by measuring bone density,

3 in other way, which ______________________________________

**Mental health problems**

**20. Do you have a psychiatric or mental illness?** 1 yes 2 no

If yes, is it: (more than one option is allowed)

1. psychosis
2. depression
3. anxiety
4. use of intoxicant agents
5. some other mental disease __________________________________________

**Other diseases detected by a physician**

**21. Diabetes** 1 yes 2 no

How old were you when the diabetes was diagnosed? _________

**22. Severe headache?** 1 yes, 2 no

What kind of headache? 1 recurrent migraine,

2 other headaches

How often does the headache represent? 1 every day or almost every day

1. a few times a week
2. a few times a month
3. one time a month
4. 5 less often

**23. Do you have a long-term illness other than the one mentioned above?** 1 yes, 2 no

What are these diseases? ––––––––––––––––––––––––––––––––––––––––––––––––––––––––––––––––––––––––––––––––––––––––––––––––––––––––––––––––––––

**Symptoms**

**24. Do you have trouble holding urine, incontinence?** 1 yes, 2 no

**Joint symptoms in more detail**

**25. Have you had pain, aches, or a movement related pain in one or more joints in the last month (30 days)?**

shoulder 1 yes, 2 no

elbow 1 yes, 2 no

wrist 1 yes, 2 no

fingers 1 yes, 2 no

hip 1 yes, 2 no

knee 1 yes, 2 no

ankle 1 yes, 2 no

foot/toes 1 yes, 2 no

**26. Have you had difficulty in walking during the last month (30 days) due to a symptom or defect in the hip?** 1 no, 2 yes

**27. Have you had trouble in walking during the last month (30 days) due a symptom or defect on the knee?**  1 no, 2 yes

**28. On a scale of 0 to 10, estimate how much harm you suffer from your back, neck, shoulder or joint problems**.

**An Inconvenience at work**

does not harm at all the worst possible harm

0............ 1………..... 2……….......3 ………......4 ……....... 5……....... 6……........7…….......8 …….......9 ……...... 10

**B** **inconvenience during leisure time**

does not harm at all the worst possible harm

0............ 1…......... 2 ….........3 ............4 …......... 5……...... 6…............7…............8 …...........9 …........... 10

**29. On a scale of 0 to 10, estimate how much pain you have experienced in the last seven days in the following parts of the body**

A Back

no pain at all the worst possible pain

0............ 1…......... 2 ….........3 ............4 …......... 5……...... 6…............7…............8 …...........9 …........... 10

B Neck

no pain at all the worst possible pain

0............ 1…......... 2 ….........3 ............4 …......... 5……...... 6…............7…............8 …...........9 …........... 10

C Shoulder

no pain at all the worst possible pain

0............ 1…......... 2 ….........3 ............4 …......... 5……...... 6…............7…............8 …...........9 …........... 10

D shoulder joints

no pain at all the worst possible pain

0............ 1…......... 2 ….........3 ............4 …......... 5……...... 6…............7…............8 …...........9 …........... 10

E Hips

no pain at all the worst possible pain

0............ 1…......... 2 ….........3 ............4 …......... 5……...... 6…............7…............8 …...........9 …........... 10

F Knees

no pain at all the worst possible pain

0............ 1…......... 2 ….........3 ............4 …......... 5……...... 6…............7…............8 …...........9 …........... 10

**Use of health care services**

**31. How many times in the last year (12 months) have you seen a doctor? It's not about going to the dentist.** (Enter 0 if you haven't visited once). ____ times

**32. How many times in the last year (12 months) have you visited a public health care nurse, a blood pressure nurse or an occupational health nurse, or a public health care nurse visited you at home?** (Enter 0, if not once). ____ times

**33. How many whole days were you absent from work due to illness or without performing your usual duties during the last year (12 months)?** (If none, please answer 0.) ____ days

**34. How many days have you been hospitalized during the last year (12 months)?** (If none, please answer 0.) ____ days

**35. When was the last time you had a health examination or a medical examination that was not done for a symptom or an illness?** (e.g. statutory workplace inspection, driving licence inspection, maternity examination)

1. in the last 1/2 year
2. 1/2 years – 1 year ago
3. 1 year – 5 years ago
4. more than 5 years ago
5. never

**Medicines**

**36. In the last 6 months, have you ever taken any prescription medicine prescribed by a doctor?** (including oral contraceptives in women under 45 years of age) 1 yes, 2 no

**37. Do you currently use any prescription medicine prescribed by your doctor?** 1 yes, 2 no

**38. What kind of drugs do you have at your disposal?** Please, report also medications taken per needed.

––––––––––––––––––––––––––––––––––––––––––––––––––––––––––––––––––––––––––––––––––––––––––––––––––––––––––––––––––––––––––––––––––––––––––––––––––––––––––––––––––––––––––––––––––––––––––––––––––––––––––––––––––––––––––––––––––––––––––––––––––––––––––––––––––––––––––––––––––

**39. In the last 6 months, have you ever used any medicine, vitamin preparation, natural medicine, herbal or herbal medicine or homeopathic product without a prescription?** 1 yes, 2 no

**40. Do you currently use such preparations?** 1 yes, 2 no

**41**. **What kind of products do you use?**

–––––––––––––––––––––––––––––––––––––––––––––––––––––––––––––––––––––––––––––––––––––––––––––––––––––––––––––––––––––––––––––––––––––––––––––––––––––––––––––––––––––––––––––––––––––––––––––––––––––––––––––––––––––––––––––––––––––––––––––––––––––––––––––––––––––––––––––––––––

**42. In the last week (7 days), have you taken any tablets, powders or other medicines?** (you can circle more than one)

yes, no

antihypertensive medicines ……………......................................................................................1

headache medications..............................................................................................................1

other painkillers........................................................................................................................1

birth control pills.......................................................................................................................1

sedative/anxiolytic medications................................................................................................1

sleeping pills.............................................................................................................................1

antidepressants........................................................................................................................1

vitamin and mineral preparations............................................................................................1

medicine for lowering colesterol..............................................................................................1

cough medicine........................................................................................................................1

hormone drugs for menopause (or post-menopause) for women………………………………….......1

**Pregnancies and childbirths**

**43. How many times have you been pregnant?** (including miscarriages and abortions) _______times

**44. How many births have you had?** (Note: Including both caesarean sections (sections) and ordinary sub-deliveries) ________ childbirth, in years _________________________

**45. Have you experienced yourself depressed after any pregnancy?**

1. no
2. yes, but I didn't need treatment
3. yes, I didn't get treatment even if I needed
4. yes, I got treatment, what was it?­­­­­­­­­­­­­­­­­­­­­­­­­­­_______________________________________________

**46. Have you had the following during any of your pregnancies?**

**a. pregnancy poisoning (toxemia)?** (Note: from proteins in urine and blood pressure elevated after the 20^th^ week of pregnancy, i.e. after the halfway period of pregnancy)

1 yes, year______

2 no

**B. Hypertension**? (> 140/90 mmHg)

1 yes, year ________

2no

**C. protein in urine?**

1 yes, year ________

2 no

**D. increased blood glucose levels**?

1 yes, year ________

2 no

**47. Have you had venous clots, or pulmonary embolism during e-pills, pregnancy or hormone replacement therapy**?

1 yes, year ______

2 no

**48. Have you or your family had venous clots or pulmonary embolism?**

1 yes, who? __________

2 no

**49. My current weight _______ kg**

**50. My current height _______ cm**

**51. My weight has**

a. increased______ kg in the past year

b. decreased______ kg in the past year

c. remained unchanged in the past year

**52. It is assumed that at best your ability to work has scored 10 points. What score would you give to your current ability to work?** (0 means that you are currently unable to work at all) a completely incapacitated working capacity at best

0............ 1…......... 2 ….........3 ............4 …......... 5……...... 6…............7…............8 …...........9 …........... 10

**Physical activity and exercise**

**53. Overall, how much do you move weekly?** Take into account all regular weekly physical exertion that lasts at least 10 minutes at a time. Circle all the options for your exercise and mark on the lines how much exercise you do (how many days a week, how many hours and minutes in total per week.) If you hardly exercise regularly weekly circle the option of "almost no regular exercise every week" and leave other options unselected.

1. Hardly any regular exercise every week

2. Slow and calm endurance exercise (= no sweating or acceleration of breathing, e.g. peaceful walking) |_____| days a week, total |_____|______| hours |_____|_____| minutes a week

3. Rapid and brisk endurance exercise (= some sweating and/or acceleration of breathing, e.g. brisk walking) |_____| days a week, total |_____|______| hours |_____|_____| minutes per week

4. Intense and strenuous endurance exercise (= intense sweating and/or acceleration of breathing, e.g. jogging or running) |_____| days a week, total |_____|______| hours |_____|_____| minutes a week

5. Muscle fitness exercise (= e.g. fitness circuit or gym training, where movements affecting different muscle groups are performed at least 8–12 times) |_____| days a week, total |_____|______| hours |_____|_____| minutes a week

6 Exercise that requires or develops balance (= e.g. tai chi, dance, exercise games, balance exercises on one foot, uneven surface or container position) |_____| days a week, total |_____|______| hours |_____|_____| minutes a week

**54. How many hours do you spend sitting on average per day (on a weekday)?** Mark 0 if none, mark only integers

During the working day, the office or similar situation |_____|____| hours |_____|_____| min a day

Watching TV or videos at home |_____|_____| hours |_____|_____| min in a day

At home in front of a computer |_____|_____| hours |_____|_____| min a day

In a vehicle (car, train, plane) |_____|_____| hours |_____|_____| min per day

In other places |_____|_____| hours |_____|_____| min in a day

**55. Do you currently smoke?**

1. not at all
2. occasionally, ________ cigarettes / week
3. yes, daily, ________ cigarettes / day

**Quality of life (SF-36)**

**Quality of life (15-D)**

**Quality of life (DEPS)**

Thank you for your answers!
